# FAMILY STRUCTURE AND DEMENTIA CAREGIVER NETWORK COMPOSITION

**DOI:** 10.1093/geroni/igae098.1316

**Published:** 2024-12-31

**Authors:** William McConnell, Katherine Haggar, Justin Hinson, Jackeline Linares, Jo Ann Bamdas, María Ortega Hernández

**Affiliations:** Florida Atlantic University, Boca Raton, Florida, United States; Florida Atlantic University, Boca Raton, Florida, United States; Florida Atlantic University, Boca Raton, Florida, United States; Florida Atlantic University, Boca Raton, Florida, United States; Florida Atlantic University, Boca Raton, Florida, United States

## Abstract

Ongoing shifts in family structure, including a growing number of adults aging without spouses or children, raise questions about who will step into a caregiving role for persons living with dementia (PLWD). Furthermore, research and interventions targeting caregivers typically limit their focus to one primary caregiver, but PLWD often rely on many interconnected informal and professional supporters. Social Network Analysis (SNA) is a valuable tool for identifying potential and actual caregivers for PLWD, including nontraditional and secondary caregivers who are often overlooked. In this presentation, we report results from an ongoing quantitative study of 250 dementia caregiver networks in the southeastern United States. Detailed SNA data were obtained from survey-based interviews with self-identified caregivers for PLWD utilizing outpatient services at a Memory Disorder Clinic or living in the community. Results indicate that PLWD in this sample are supported by 7.7 caregivers, on average. While traditional caregivers such as spouses and adult children remain common, other relatives including siblings, nieces/nephews, and grandchildren comprise nearly 20% of all informal caregivers. Non-kin friends comprise an additional 20% of informal caregivers on their own. We describe how family, extended family, and non-kin caregivers in the networks differ in terms of their participation rates and task-specific roles. We also present multivariate analyses examining how caregiver network composition changes based on disease severity and the availability of potential caregivers within families/households. We conclude by discussing implications of SNA for understanding caregiving in the context of shifting family norms.

